# APP ENGAGEMENT IN TECHNOLOGY-SUPPORTED SPEECH-LANGUAGE THERAPY FOR PRIMARY PROGRESSIVE APHASIA

**DOI:** 10.1093/geroni/igae098.4376

**Published:** 2024-12-31

**Authors:** Ollie Fegter, Alfred Rademaker, Matthew Bona, Angela Roberts, Emily Rogalski

**Affiliations:** Northwestern University Feinberg School of Medicine, Chicago, Illinois, United States; Northwestern University Feinberg School of Medicine, Chicago, Illinois, United States; University of Chicago, Chicago, Illinois, United States; Western University, London, Ontario, Canada; University of Chicago, Chicago, Illinois, United States

## Abstract

As the global population ages, dementia prevalence is increasing. Primary Progressive Aphasia (PPA) is a clinical dementia syndrome characterized by progressive language decline. Access to care for individuals living with PPA is limited by a shortage of qualified clinicians and evidence-based interventions. While technology-supported interventions have the potential to overcome this barrier, there has been no systematic exploration of factors affecting web application use in this population. This study aimed to evaluate feasibility and acceptability and identify clinical and demographic factors affecting app usage in individuals with PPA. Web application data were analyzed from participants (N=95) of Communication Bridge-2, an NIH stage 2 randomized controlled trial of speech-language therapy for PPA (NCT03371706). Participants were encouraged to complete app-based practice exercises five days per week over the 12-month intervention. Data-driven user-type clustering determined engagement groups based on number of logins. Multinomial logistic regression identified demographic and clinical factors that differed between groups. On average, participants completed 13.7 (SD = 5.95) home practice exercises 3.99 days per week (SD = 1.19) and logged into the app 5.88 times per week (SD = 1.29). Older age and increased education significantly predicted increased app engagement (p <.05), even when accounting for work status; other clinical and demographic factors were nonsignificant. All participants logged into and completed practices exercises on the app at least weekly, demonstrating high feasibility and acceptability. These findings suggest that technology-supported interventions may be a valuable tool to improve access to evidence-based care to individuals with PPA and related conditions.

